# Diosgenin promotes antitumor immunity and PD-1 antibody efficacy against melanoma by regulating intestinal microbiota

**DOI:** 10.1038/s41419-018-1099-3

**Published:** 2018-10-10

**Authors:** Mengxue Dong, Zhefeng Meng, Kudelaidi Kuerban, Feilong Qi, Jiayang Liu, Yuxi Wei, Qian Wang, Shanshan Jiang, Meiqing Feng, Li Ye

**Affiliations:** 1 Department of Microbiological & Biochemical Pharmacy, School of Pharmacy, Fudan University, 201203 Shanghai, China; 2 Minhang Branch, Zhongshan Hospital, Fudan University, 201199 Shanghai, China; 3 Department of Pharmacy, Shanghai Ninth People’ s Hospital, Shanghai Jiao Tong University School of Medicine, 200011 Shanghai, China

## Abstract

Diosgenin, a natural steroidal saponin, can exert antitumor effect by regulating immune function and improving intestinal microbiota. The response to anti-PD-1 immunotherapy is associated with intestinal microbiota and effector T cells in tumor microenvironment. We hypothesize that the modulation of diosgenin on intestinal microbiota can facilitate antitumor immunity and the therapeutic efficacy of PD-1 antibody. In melanoma-bearing C57BL/6 mice, we observed that the anti-melanoma effect of diosgenin relied more on antitumor immunity than direct tumor inhibition activity evidenced by obvious CD4^+^/CD8^+^ T-cell infiltration and IFN-γ expression in tumor tissues, and it could improve the compositions of intestinal microbiota. Antibiotics impaired the therapeutic efficacy and immunity responses of diosgenin through disturbing intestinal microbiota, indicating the importance of intestinal microbiota in diosgenin’s in vivo antitumor activity. More importantly, the combined administration of PD-1 antibody with diosgenin aggravated the tumor necrosis and apoptosis by eliciting augmented T-cell responses. Taken together, diosgenin can be used as a microecological regulator to induce antitumor immunity and improve the efficacy of immune checkpoint antibody, making it more suitable for the treatment of malignant tumors.

## Introduction

Tumor immunotherapy has aroused general concern due to its capacity to enhance the host’s immune surveillance to recognize tumor cells, and then make use of autoimmune function to eradicate tumor and produce sustained antitumor immune response^1^. In the process of tumor progression, the immune activity of T cells is dampened by upregulation of the immune checkpoint molecules such as PD-1/PD-L1 and CTLA-4 in the tumor microenvironment, resulting in tumor immune escape. Consequently, blockade of immune checkpoint has become a research focus of current tumor immunotherapy^2,3^.

Recently, immunotherapy represented by immune checkpoint inhibitors targeting the PD-1/PD-L1 axis and CTLA-4 has delivered favorable results in many solid tumors^4^. In advanced melanoma patients, treatment with PD-1 antibody pembrolizumab prolonged progression-free survival (PFS) and overall survival with less adverse events^5^. Among patients with non-small-cell lung cancer and a high tumor mutational burden, significantly longer PFS occurred with combination therapy of PD-1 antibody nivolumab and CTLA-4 antibody ipilimumab than with chemotherapy^6^. Besides, pembrolizumab was approved by FDA for the treatment of solid tumor with high microsatellite instability or defective mismatch repair, and became the first “broad-spectrum anticancer drug” based on common biomarkers rather than specified tumor locations^7^. This has accelerated single-agent or multi-drug combination therapy of immune checkpoint inhibitors represented by PD-1 antibodies. Despite all of these advantages, unfortunately only about 25% of patients with solid tumors can benefit from such immunotherapy. It emphasizes that how to improve the clinical response rate of patients to immune checkpoint antibodies is the main obstacle.

It is thought that interpatient heterogeneity in the clinical response to immune checkpoint inhibitors is closely interrelated with cancer-cell-autonomous cues, tumor-microenvironmental factors, and host-related influences^8^. Among them, intestinal microbiota as a promoter of tumor immunotherapy has now become a topical issue^9,10^. Numerous researches suggest that regulating the microbiota may modulate cancer immunotherapy and promote the efficacy of immune checkpoint antibodies^11–13^. There are two studies that highlight the key role of the intestinal microbiota in mediating tumor response to immune checkpoint inhibitors: (1) The antitumor efficacy of CTLA-4 antibody depended on the immunostimulatory effect of intestinal microbiota, in which distinct bacterial species could promote the maturation of intratumoral dendritic cells and induced interleukin-12-dependent Th1 immune response^14^. (2) Intestinal *Bifidobacterium* could facilitate the dendritic cell function that in turn enhanced tumor-specific CD8^+^ T-cell priming, and accumulation in the tumor microenvironment subsequently promoted the efficacy of PD-L1 antibody against melanoma^11^. Meanwhile, prior or concomitant modulation of the intestinal microbiota can optimize treatment outcomes of immune checkpoint inhibitors^8^. This phenomenon has been further confirmed in human cancer patients. Three recent published studies reported that intestinal commensal microbiome was closely related to the efficacy of anti-PD-1 immunotherapy in melanoma and epithelial cancer patients^15–17^.

Diosgenin is a natural steroidal saponin derived from the genus *Dioscorea*. As a traditional Chinese medicine (TCM), it can be used for controlling hyperlipidemic, hyperglycemic, and cardiovascular diseases, and even has immunomodulatory and antitumor functions^18–20^. It has been reported that diosgenin and its derivatives possessed the ability to remedy melanoma, leukemia, and lung cancer by inducing cell cycle arrest, cell differentiation, and apoptosis^21–23^. Specifically, diosgenin can significantly inhibit the growth of sarcoma-180 tumor by improving both specific and nonspecific cellular immune effects^23^, increase the expressions of IFN-γ and antigen-specific IgG_2a_, and enhance immune response mediated by Th1 cells^24^. It was recently found that diosgenin has similar activity to prebiotics. Oral diosgenin can notably upregulate the composition of intestinal lactic acid bacteria in mice, which breaks the normal concept that prebiotics are mostly oligosaccharides^25^.

In this study, we aim to determine whether the antitumor immune effect caused by diosgenin is achieved through regulating the intestinal microbiota in melanoma-bearing immunocompetent mice. Moreover, taking into consideration that the efficacy of PD-1 antibody was influenced by intestinal microbiota and positively correlated with the number of effector T cells in the tumor microenvironment^26,27^, this study further explores whether the diosgenin-induced intestinal microbiota modulation and antitumor immunity can improve the therapeutic efficacy of PD-1 antibody.

## Results

### Diosgenin exerted anti-melanoma activity in vitro

**Fig. 1:**
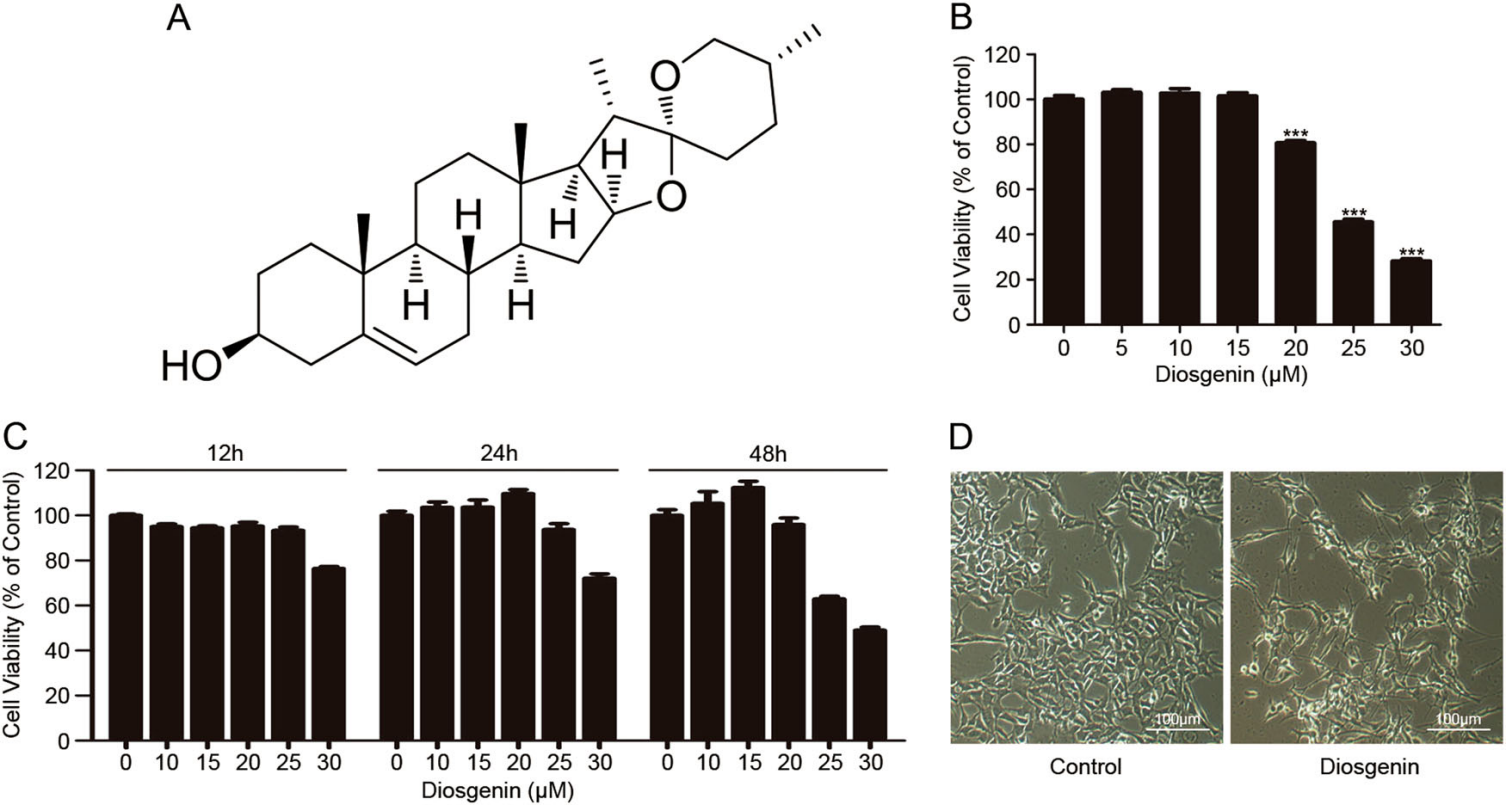
Diosgenin induced cytotoxicity in B16F10 cells. **a** The structure of diosgenin. **b** B16F10 cells were treated with various concentrations of diosgenin for 48 h, and then MTT assay was applied to measure the cell viability (IC_50_ of diosgenin = 25 μM). All these data were represented as mean ± SD (***P < 0.001). **c** B16F10 cells were exposed to different concentrations of diosgenin for indicated times. Cell viability was measured by MTT assay. **d** B16F10 cells were administered with 25 μM diosgenin for 48 h and the morphological alterations of cells were observed by an inverted microscope

The structure of diosgenin is shown in Fig. 1a. 3-(4, 5-dimethylthiazol-2-yl)-2, 5-diphenyltetrazolium bromide (MTT) assay was used to determine the cytotoxicity of diosgenin by detecting the cell viability. After exposure to different concentrations of diosgenin for indicated times, the B16F10 melanoma cell viability decreased in a dose- and time-dependent manner within the tested range (Fig. 1b, c). Meanwhile, cells displayed visible morphological changes after treatment with diosgenin, including cell shrink, fragmentation, and reduced number of cells (Fig. 1d).

Altogether, diosgenin induced cell viability reduction and obvious morphological alteration in melanoma cells, which indicated the anti-melanoma effect of diosgenin in vitro.

### In vivo anti-melanoma effect of diosgenin was associated with antitumor immunity

**Fig. 2:**
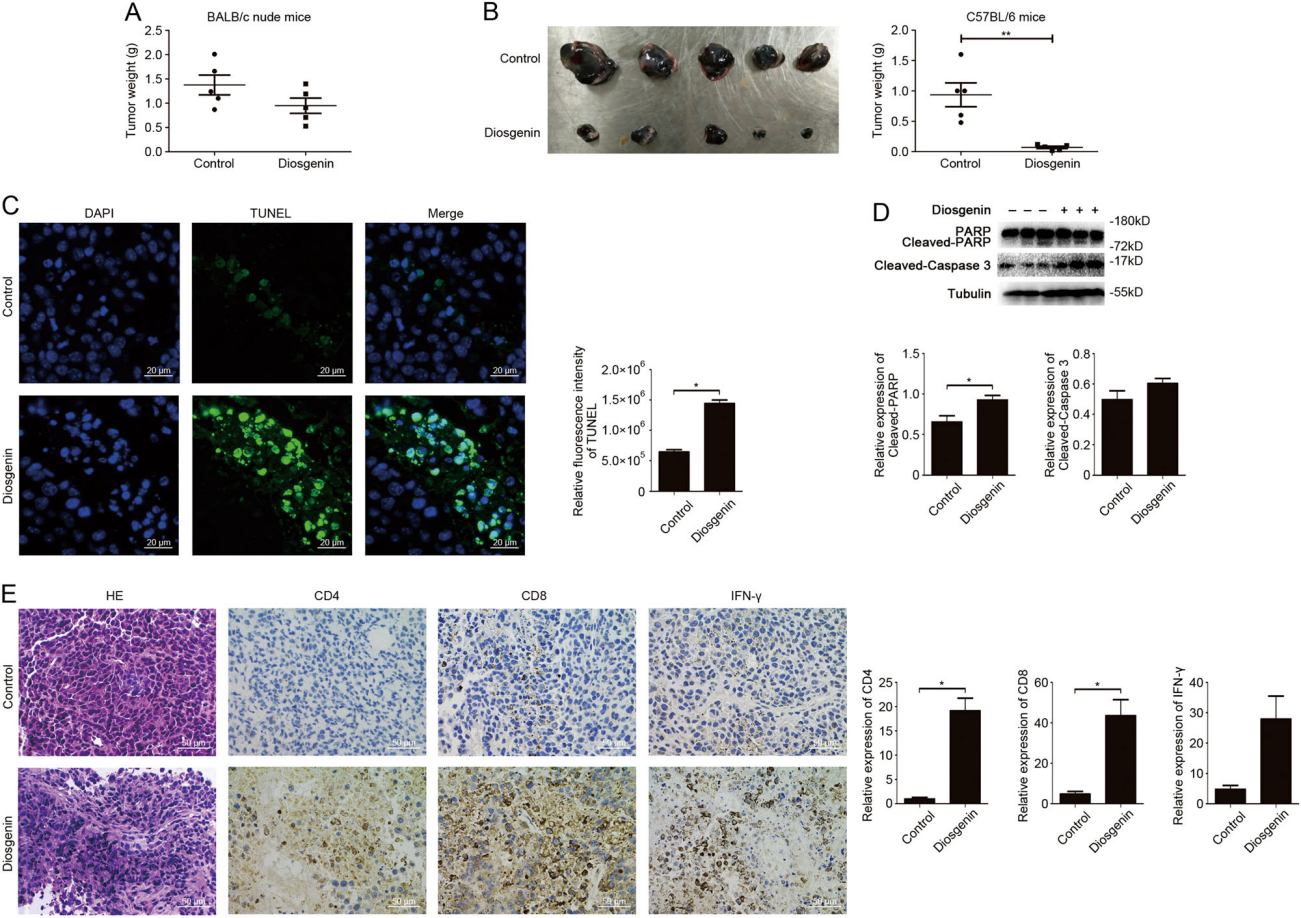
In vivo anti-melanoma activity of diosgenin dependent on its antitumor immunity. The mice were subcutaneously injected with 2×10^5^ of B16F10 cells. **a** The tumor weight of nude mice daily treated with normal saline (control) and diosgenin (20 mg/kg) respectively for 2 weeks. The results were represented as means ± SD. **b**−**e** The tumor-bearing C57BL/6 mice were treated with or without diosgenin (20 mg/kg) everyday. After administration for 2 weeks, the tumors were excised (**b**, left), and the tumor weight was shown in (**b**, right). **c** Apoptosis of tumor tissues from C57BL/6 mice was assayed with TUNEL and observed under fluorescence microscope. The quantitation analysis of apoptotic tumor cells was performed by ImageJ software and presented in bar charts. **d** The expression levels of PARP, cleaved-PARP, and cleaved-caspase 3 in tumor tissues from C57BL/6 mice were checked by western blot analysis. Densitometric values were quantified by ImageJ software. **e** Representative H&E staining images of tumor tissues were shown in the left panel, and the expressions of CD4, CD8, and IFN-γ in tumor tissues were detected by immunohistochemistry. The quantitation analysis was performed by ImageJ software. All these data were presented as mean ± SD (*P < 0.05, **P < 0.01)

In the mice model of subcutaneous tumor produced by B16F10 melanoma cells, the antitumor effect was initially evaluated after diosgenin administration by measuring the tumor weight. As shown in Fig. 2a, diosgenin slowed down the tumor growth, but the inhibition rate was only 31.1% as compared to the control in BALB/c nude mice. The inhibition rate was calculated as [(*A*−*B*)/*A*] × 100%, where *A* and *B* were the average tumor weights of the negative control and experimental groups respectively. Surprisingly, the tumor weight reduced more obviously in diosgenin-treated immunocompetent C57BL/6 mice with inhibition rate of 92.5% (Fig. 2b). TUNEL assay displayed a significantly large number of apoptotic cells in tumor tissues harvested from C57BL/6 mice treated with diosgenin (Fig. 2c). Western blot analysis also illustrated that the cleavage of apoptosis-related proteins such as PARP and caspase-3 were both upregulated (Fig. 2d), indicating that diosgenin can induce more apoptosis in tumor tissues. Moreover, hematein-eosin (H&E) staining detected extensive necrosis within the tumor in diosgenin-treated cohort (Fig. 2e). Because of the remarkable difference in anti-melanoma effects of diosgenin between immunodeficient mice and immunocompetent mice, we speculated that diosgenin might induce antitumor immunity besides direct tumor inhibition activity in vivo. Immunohistochemical analysis of tumor tissues was then conducted to explore whether diosgenin can trigger immune effect in tumor-bearing C57BL/6 mice. Compared with the control, the expressions of CD4, CD8, and IFN-γ were positive in diosgenin-treated cohort, suggesting the infiltration of CD4^+^ T and CD8^+^ T cells as well as augmented immune response in tumor tissues (Fig. 2e).

These results demonstrated that in vivo anti-melanoma effect of diosgenin relied more on antitumor immune effect than direct tumor inhibition activity.

### The modulation of intestinal microbiota was triggered by diosgenin

**Fig. 3:**
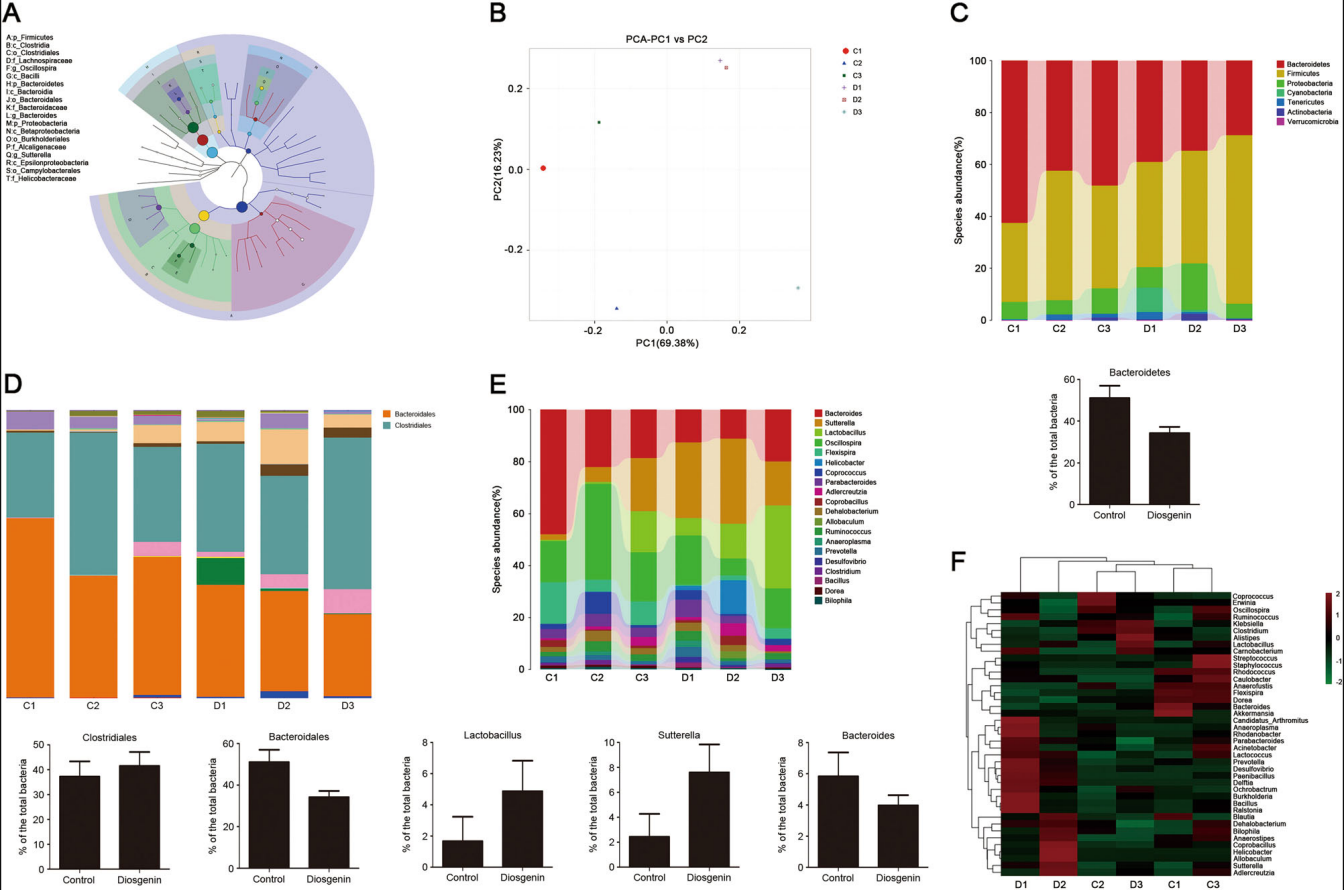
Diosgenin had a regulatory effect on intestinal microbiota in C57BL/6 mice. The C57BL/6 mice were administered by gavage with normal saline (control) or diosgenin (20 mg/kg) everyday; the feces of mice were collected after 2 weeks to detect the intestinal microbiota. C1, C2, and C3 belonged to the control group, D1, D2, and D3 belonged to the diosgenin-treated group. **a** The classification tree of sample population based on GraPhlAn. **b** Two-dimension ordination graph of PCA analysis, in which distances between the samples represented the differences of them. **c**−**e** The community taxonomic composition and abundance distribution map from phylum to genus. The taxonomic units were distinguished by different colors, and the length of the column represented the relative abundance of each unit. **c** At the phylum level, the abundance of Bacteroidetes was counted. **d** At the order level, the abundances of Clostridiales and Bacteroidales were analyzed. **e** According to the level of genus, the abundances of *Lactobacillus*, *Sutterella*, and *Bacteroides* were respectively analyzed. All the results were presented as mean ± SD. **f** The heatmap with log_10_-transformation of relative abundance at the genus level in the intestine that was indicated from green (less) to red (more)

Relative abundance of each Operational Taxonomic Unit (OTU) and other taxonomic levels (from phylum to genus) was evaluated for each sample. The number of sequences clustered within each OTU (or other taxonomic levels) was converted to a fraction representing the relative contribution of each feature to each individual. The classification tree showed the hierarchical relationship among all taxonomic units (represented by nodes) from phylum to genus (from inner ring to outer ring) in the sample population, and the taxons of the top 20 relative abundances were identified by letters (Fig. 3a). Principal component analysis (PCA) maximized the distances between the samples in the coordinate system to reflect the actual differences. As shown in Fig. 3b, the intestinal microbial community structure notably changed after diosgenin administration. In the community taxonomic composition and abundance distribution map at phylum level, the column of Bacteroidetes was shorter than that in the control after treatment with diosgenin, indicating that Bacteroidetes were downregulated (Fig. 3c). Meanwhile, based on bacterial order composition, the increase of Clostridiales and the reduction of Bacteroidales were observed (Fig. 3d). It can also be found that *Lactobacillus* and *Sutterella* were obviously upregulated as well as *Bacteroides* were downregulated at genus level (Fig. 3e). For heatmap, log_10_-transformation was applied on the relative abundance data matrix, which allowed visualizing similarities or differences between samples. After exposure to diosgenin, microbial community differences became smaller, making the species composition have the higher degree of uniformity (Fig. 3f).

In summary, these results illustrated that diosgenin, as a modulator, could change the composition of the intestinal microbiota in C57BL/6 mice.

### Diosgenin induced anti-melanoma activity and immune effect by regulating intestinal microbiota

**Fig. 4:**
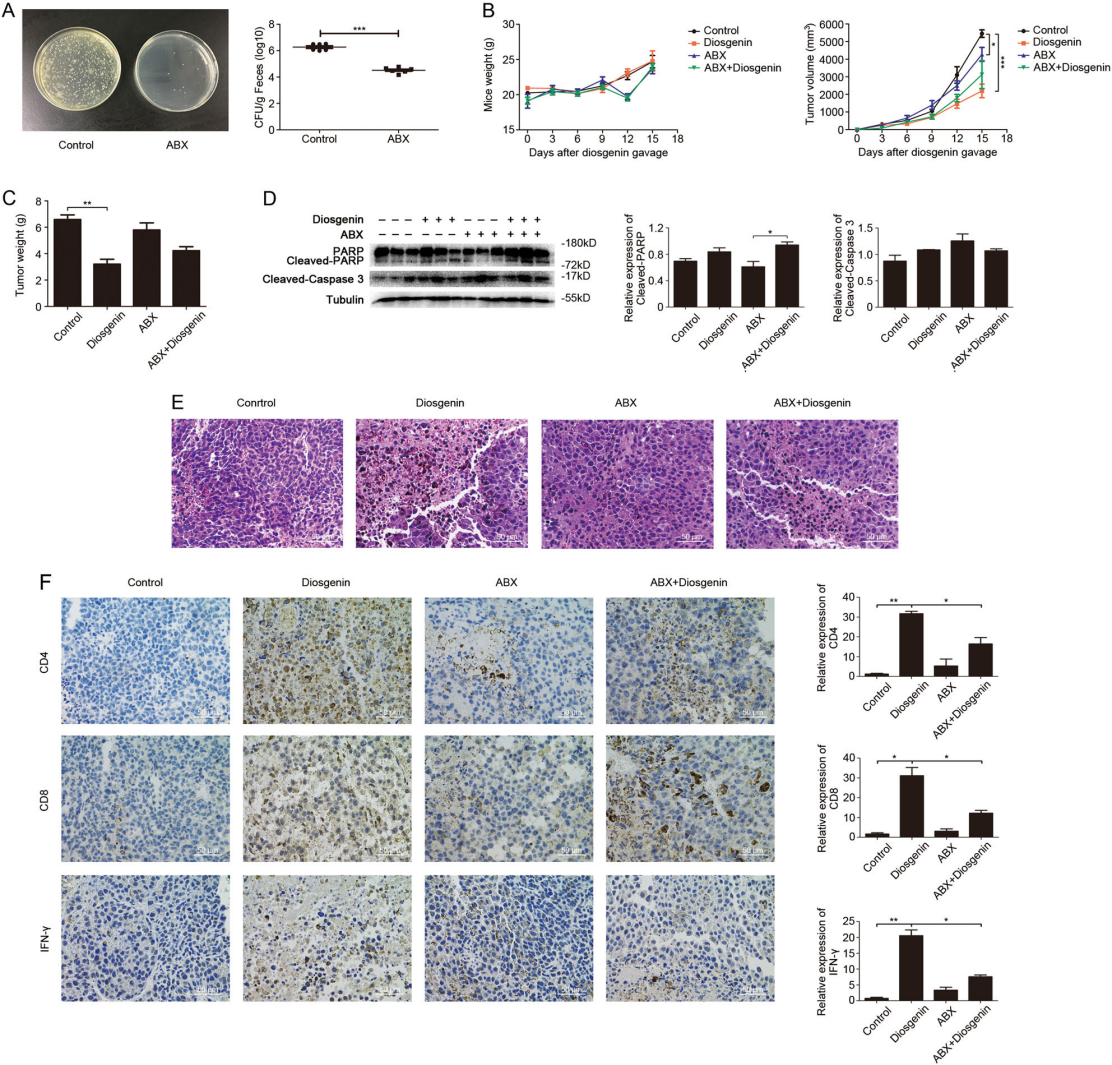
Diosgenin triggered anti-melanoma immune effect by modulating intestinal microbiota in tumor-bearing C57BL/6 mice. The C57BL/6 mice received antibiotic cocktail (ABX) 2 weeks before tumor inoculation. After subcutaneously injected with 1×10^6^ of B16F10 cells, the mice were daily treated with or without diosgenin (20 mg/kg). **a** The feces of mice were cultivated on LB solid plates (left) post-ABX administration, and the number of bacterial colonies was shown in the right panel. **b** The mice weight and the tumor volume of four groups were detected throughout the experiment. After mice were sacrificed, the tumor weight was measured (**c**). **d** The expression levels of PARP, cleaved-PARP, and cleaved-caspase 3 in tumor tissues were assessed by western blot analysis. Densitometric values were quantified by ImageJ software. **e** Representative H&E staining images of tumor tissues from each group. **f** Immunohistochemical analysis of tumor tissues, and the expression levels of CD4, CD8, and IFN-γ were observed under microscope. The quantitation analysis was performed by ImageJ software and presented in bar charts. All these data were represented as mean ± SD (*P < 0.05, **P < 0.01, ***P < 0.001)

To explore whether the intestinal microbiota affects the antitumor immune effect of diosgenin in vivo, C57BL/6 mice received antibiotic cocktail (ABX), and the varieties and numbers of their intestinal microbiota were visibly changed after 2 weeks of this treatment (Fig. 4a). In subcutaneous melanoma models, the mice weight increased steadily in the control and diosgenin-treated cohorts, and the weight of the mice after antibiotic treatment tended to increase with slight fluctuations, indicating that the diosgenin and ABX were safe at the administration dosage. Meanwhile, among the four groups the tumor volume in diosgenin-treated cohort increased most slowly from day 9 till the remaining experimental period (Fig. 4b). When mice were sacrificed on day 15 and the tumors were resected, the tumor weight of cohort treated with diosgenin was 3203.33 ± 641.43 mg versus 6586.67 ± 603.52 mg of the control, and the tumor weight of the cohort cotreated with diosgenin and ABX was 4220.00 ± 540.65 mg (Fig. 4c). Afterwards, western blot analysis was performed on harvested tumor tissues to analyze apoptosis. As shown in Fig. 4d, the cohort treated with diosgenin as well as cohort cotreated with diosgenin and ABX both increased the cleavage of PARP and caspase-3, but there were no significant differences between these two. Histological and immunohistochemical analysis displayed that, in tumor-bearing mice administered with diosgenin, the tumor necrosis and CD4^+^/CD8^+^ T-cell infiltration increased along with an enhancement of IFN-γ expression in tumor tissues. However, ABX treatment could greatly cut down all of these growth trends mentioned above (Fig. 4e, f).

All of these results indicated that the integrity of the intestinal microbiota was crucial for diosgenin to induce the antitumor activity and immune effect in vivo.

### Diosgenin enhanced the efficacy of PD-1 antibody by eliciting immune effect

**Fig. 5:**
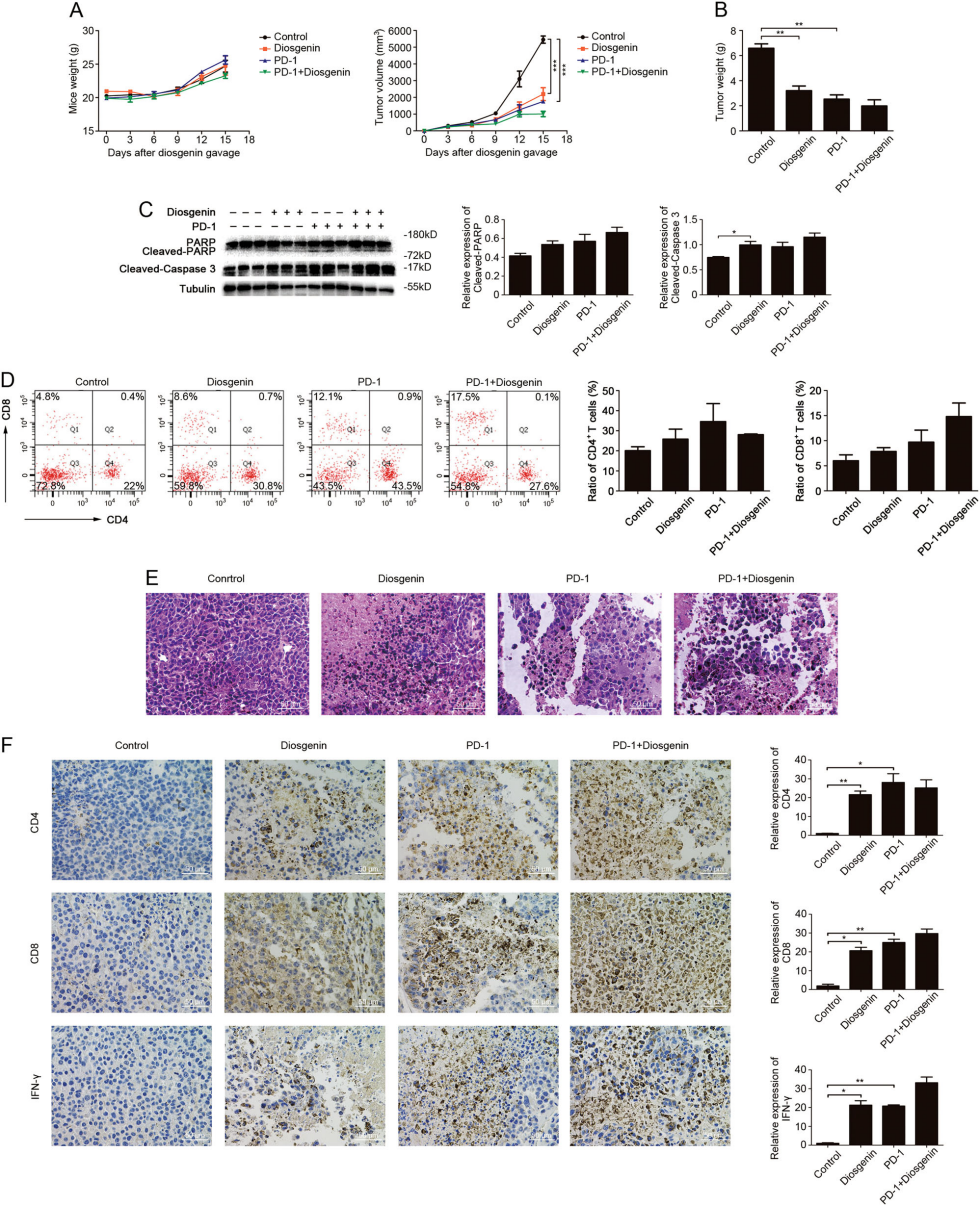
Combined administration of diosgenin and PD-1 mAb achieved the best antitumor effect. After subcutaneously injected with 1×10^6^ of B16F10 cells, the C57BL/6 mice were daily treated with or without diosgenin (20 mg/kg), and then injected intraperitoneally with PD-1 mAb on days 6, 9, and 12 post-tumor implantation. **a** The mice weight and the tumor volume of four groups were detected throughout the experiment. **b** After the whole experiment, the tumor weight was measured. **c** The expression levels of PARP, cleaved-PARP, and cleaved-caspase 3 in tumor tissues were assessed by western blot analysis. Densitometric values were quantified by ImageJ software. **d** The number of CD4^+^/CD8^+^ T cells in tumor tissues was analyzed by flow cytometry, and the percentage of these cells was presented in bar charts as mean ± SD. **e** Representative H&E staining images of tumor tissues from each group. **f** The expression levels of CD4, CD8, and IFN-γ in tumor tissues were determined by immunohistochemistry, and the quantitation analysis was performed by ImageJ software. All these data were presented as mean ± SD (*P < 0.05, **P < 0.01, ***P < 0.001). PD-1 indicates PD-1 monoclonal antibody

The experiments above confirmed that diosgenin could regulate intestinal microbiota and induce immune effect, and it is reported that the efficacy of PD-1 antibody had a certain correlation with both of them. Thus, we further evaluated whether diosgenin could enhance the response rate and the efficacy of PD-1 antibody. B16F10 subcutaneous tumor model was established, and then the C57BL/6 mice were treated with PD-1 mAb 6 days later. As shown in Fig. 5a, the mice weight continued to increase steadily throughout the experiment in all cohorts, and the tumor volume increased more slowly from day 9 till the remaining experimental period in the cohort cotreated with diosgenin and PD-1 mAb as well as the cohort treated with diosgenin or PD-1 mAb alone than the control. Then, mice were sacrificed and tumors were resected after 15 days of treatment. Mean tumor weight of the cohort cotreated with diosgenin and PD-1 mAb was 1980.00 ± 861.22 mg versus 3203.33 ± 641.43 mg of the cohort treated with diosgenin alone, and tumor weight of the cohort treated with PD-1 mAb alone was 2530.00 ± 584.04 mg (Fig. 5b). Western blot analysis showed that combined administration of diosgenin and PD-1 mAb increased the cleavage of PARP and caspase-3, indicating that apoptosis was enhanced in tumor cells (Fig. 5c). After planned administration, the number of tumor-infiltrating CD4^+^ and CD8^+^ T cells increased from 22 to 30.8% and from 4.8 to 8.6% respectively in diosgenin-treated cohort, while that increased to 27.6 and 17.5% respectively in combined administration cohort (Fig. 5d). Histological and immunohistochemical analysis further revealed that in tumor-bearing mice treated with diosgenin and PD-1 mAb, the tumor necrosis, CD4^+^/CD8^+^ T-cell infiltration, and IFN-γ expression in tumor tissues were all upregulated more obviously than other cohorts (Fig. 5e, f).

Collectively, these results suggested that diosgenin could improve the efficacy of PD-1 antibody by inducing immune effect.

## Discussion

In recent years, plant saponins from diverse sources have attracted much attention as immune adjuvants^28^. The saponin components from *Acacia concinna* can effectively induce the humoral and cellular immune responses by enhancing the proliferation of lymphocyte and activation of effector T cells^29^. Platycodin D2 can dramatically increase the expressions of IL-2 and IFN-γ, and then activate the Th1 immunoreaction^30^. The semisynthetic saponin derivative GPI-0100 can activate Th1 immunity and IFN-γ expression in preclinical trials^31^. Diosgenin is a main component of TCM Di’ao xinxuekang that is mainly used for the treatment of coronary heart disease and angina, and its antitumor activity and ability to cause specific or nonspecific cellular immune effects have been demonstrated^20,23^.

In the present study, we first observed that diosgenin could inhibit the growth of B16F10 melanoma cells in vitro and in vivo as well as alter morphological features of tumor cells. However, its tumor suppression effect in immunocompetent C57BL/6 mice was superior to that in BALB/c nude mice. It meant that diosgenin not only directly exerted antitumor activity, but also was likely to enhance tumor killing effect by inducing the antitumor immunity of mice. Our experiments proved that CD4^+^/CD8^+^ T-cell infiltration and IFN-γ expression in tumor tissues of C57BL/6 mice significantly increased after diosgenin treatment, indicating the activation of the immune system^32^. Simultaneously, diosgenin could trigger visible necrosis and apoptosis in tumor tissues from C57BL/6 mice. Therefore, we conclude that diosgenin’s in vivo anti-melanoma activity mainly depends on its antitumor immunity induction effect.

The concept that diosgenin has similar activity to prebiotics was further confirmed by its ability to improve the intestinal microecology of Type 2 diabetic mice^33^. Consequently, we investigated the diversities and the structural features of intestinal microbiota in C57BL/6 mice based on the sequencing results on V4 region of 16S rDNA. After diosgenin administration, the abundances of the *Lactobacillus* genus in the Firmicutes phylum and the *Sutterella* genus in the Proteobacteria phylum were significantly upregulated, whereas the abundance of the *Bacteroides* genus in the Bacteroidetes phylum was downregulated. It has been reported that intestinal probiotics represented by *Lactobacillus* can inhibit tumor formation, and the metastatic melanoma patients with high abundance of *Lactobacillus* were sensitive to the treatment of PD-1 antibody^17,34^. *Sutterella* were positively associated with proinflammatory immune effects^35^. *Bacteroides* can induce regulatory T cells, its downregulation may imply the elimination of immunosuppression^36^. Besides, we found that diosgenin could increase the abundance of Clostridiales order and reduce the abundance of Bacteroidales order. In melanoma patients, the high abundance of intestinal Clostridiales represented better sensitivity to PD-1 antibody treatment, and the high abundance of Bacteroidales showed insensitivity to this therapy^16^. These results suggest that diosgenin can modulate the intestinal microbiota in C57BL/6 mice, and the diversities and the overall structural changes of intestinal microbiota triggered by diosgenin may be related to its antitumor immune effect.

Recent clinical investigation confirmed that antibiotics could induce intestinal microbiota dysbiosis and then affect antitumor immune responses and effectiveness of immune checkpoint inhibitors^37^. To further verify the necessity of intestinal microbiota on the antitumor immunity triggered by diosgenin, we used the antibiotic cocktail method to disturb the intestinal microbiota of C57BL/6 mice, and then observed the therapeutic efficacy of diosgenin on B16F10 melanoma^14^. It displayed that ABX destroyed the intestinal microbiota, which subsequently weakened the tumor suppression activity of diosgenin evidenced by increased tumor weight and growth speed. Diosgenin-induced tumor necrosis was also greatly alleviated by ABX cotreatment. Similar downtrend of CD4^+^/CD8^+^ T-cell infiltration and IFN-γ expression in tumor tissues of ABX cotreated mice was detected by immunohistochemical analysis. These results indicate that the damage of intestinal microbiota triggered by ABX indeed impaired the antitumor immunity and the therapeutic efficacy of diosgenin in vivo, which confirmed our hypothesis that diosgenin-induced antitumor immune effect was mediated by the intestinal microbiota. Nevertheless, ABX treatment had little effect on the apoptosis of tumor cells induced by diosgenin, and the group treated with ABX alone showed a weak CD4^+^/CD8^+^ T-cell infiltration. It has been reported that the ABX-treated mice had relatively higher frequency of effector T cells in gut wall or spleen and higher circulating level of IFN-γ along with lower IL-10 level to increase the susceptibility to inflammation^38,39^. Thus, it is better to apply germ-free mice to confirm the role of intestinal microbiota in the antitumor effect of diosgenin in further studies.

Moreover, intestinal microbiota can improve therapeutic efficacy of CpG oligonucleotides, platinum compounds, and cyclophosphamide by regulating the function of some tumor-microenvironmental immune cells and facilitating tumor infiltration of effector T cells, which ensures a longer PFS for the patients with advanced lung and ovarian cancers^12,13,40^. Blockade of immune checkpoint can strengthen and maintain endogenous antitumor effect, keeping the tumor under sustained control^41^. The clinical activity of PD-1/PD-L1 antibodies is positively correlated with the integrity of intestinal microbiota and the number of effector T cells in the tumor microenvironment, and modulation of intestinal microbiota composition may be a promising strategy to improve clinical benefit from PD-1/PD-L1 antibodies^27,37,42^. So, we further explored whether diosgenin could promote the therapeutic efficacy of PD-1 antibody by regulating intestinal microbiota to induce antitumor immunity in C57BL/6 mice. Our experiments proved that the combined administration of diosgenin and PD-1 mAb enhanced the tumor growth inhibition as well as aggravated the tumor necrosis and apoptosis. We also found that the CD4^+^/CD8^+^ T-cell infiltration and IFN-γ expression in tumor tissues were distinctly upregulated through this combination treatment. These results illustrate that diosgenin can improve the response rate and the therapeutic efficacy of PD-1 antibody by eliciting immune effect.

**Fig. 6:**
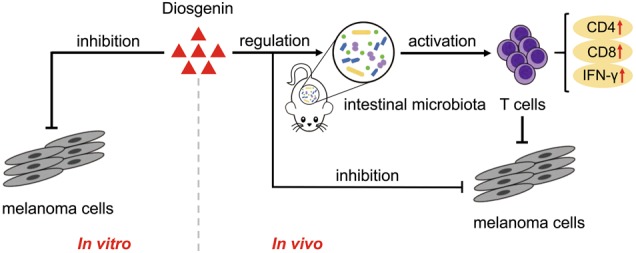
Overview of anti-melanoma effect induced by diosgenin in vitro and in vivo. Diosgenin can inhibit the growth of B16F10 melanoma cells in vitro. In vivo, the anti-melanoma effect of diosgenin is mainly dependent on antitumor immunity which is induced by its regulation of intestinal microbiota in C57BL/6 mice

In conclusion, our research proves that diosgenin can trigger growth inhibition of B16F10 melanoma cells in vitro as well as tumor necrosis, apoptosis, and activation of antitumor immunity in vivo. Meanwhile, diosgenin has the regulatory effect on intestinal microbiota in mice, and its antitumor immune effect is associated with intestinal microbiota (Fig. 6). Furthermore, diosgenin can enhance the therapeutic efficacy of PD-1 antibody by eliciting T-cell immune effect. This is the first report to study the effects of diosgenin on antitumor immunity and the therapeutic efficacy of immune checkpoint antibody based on the intestinal microecology. These results have important implications for the application of TCM in tumor immunotherapy and combination therapy with immune checkpoint inhibitors.

## Materials and methods

### Regents and antibodies

Diosgenin with a purity of more than 98% was obtained from Nanjing Spring & Autumn Biotech Co., Ltd (Jiangsu, China). Other reagents were purchased as follows: MTT (Sigma-Aldrich), InVivoMAb anti-mouse PD-1 (clone 29F.1A12, BioXCell), vancomycin, ampicillin, neomycin, and metronidazole (Dalian Meilun Biotech Co., Ltd), RIPA Lysis Buffer and phenylmethanesulfonyl fluoride (PMSF) (Beyotime Biotechnology), xylene and H_2_O_2_ (Sinopharm Chemical Reagent Co., Ltd.), 10* Proteinase K stock solution and hematoxylin (Google Biotechnology), Diaminobenzidine (DAB) Developer (DAKO), EDTA (PH9.0) Antigen Repair Buffer (Servicebio), TUNEL Assay kit (Roche), MoBio PowerSoil® DNA Isolation Kit (MoBio), Quant-iT PicoGreen dsDNA Assay Kit (Invitrogen), TruSeq Nano DNA LT Library Prep Kit and MiSeq Reagent Kit V3 (Illumina). The primary antibodies including CD4, CD8, and IFN-γ as well as the horseradish peroxidase (HRP)-conjugated secondary antibody against rabbit for immunohistochemistry were purchased from Servicebio, Inc (Boston, USA). Anti-mouse antibodies CD4 (clone GK1.5) and CD8a (clone 53-6.7) were purchased from BioLegend, and CD3e monoclonal antibody (clone 145-2C11) was purchased from eBioscience for flow cytometry analysis.

### Cell lines and culture conditions

The C57BL/6-derived melanoma cell line B16F10, purchased from Cell Bank of Chinese Academy of Sciences, Shanghai Branch (Shanghai, China), was cultured in Dulbecco's Modified Eagle Medium (DMEM) (Corning) containing 10% fetal bovine serum (Gibco), 100 IU/mL of penicillin and 100 μg/mL of streptomycin (Beyotime Institute of Biotechnology) at 37 °C in a humidified atmosphere of 5% CO_2_ incubator.

### Cytotoxicity on B16F10 cells in vitro

About 1×10^4^ cells were planted in 96-well plates and allowed to attach overnight, then exposed to different concentrations of diosgenin for indicated times. Then, the incubation of these cells with MTT (0.5 mg/mL) was implemented at 37 °C and 100 μL Dimethyl Sulfoxide (DMSO) was added to dissolve the formazan crystals. The optical density was measured at the wavelength of 570 nm by microplate reader.

### Microscopy and photography

About 5×10^3^ cells were seeded into six-well plates and then incubated with indicated concentration of diosgenin. Cells were observed by using an inverted microscope (Nikon, Japan) after incubation for 48 h.

### Animals and tumor models

All animal experiments were carried out in compliance with the standards approved by Animal Ethical Committee of School of Pharmacy at Fudan University. The 6-week-old female SPF BALB/c nude mice and C57BL/6 mice (18–20 g) were subcutaneously injected with appropriate density of B16F10 cells suspended in normal saline to establish tumor models.

### Diosgenin administration and PD-1 mAb immunotherapy in vivo

The well-established mice were randomly divided into the indicated groups, in which the mice were daily treated with diosgenin (20 mg/kg, 0.2 mL) by gavage on the second day post-tumor implantation, and normal saline treatment was served as the control. For combination therapy experiments, mice were additionally injected intraperitoneally with 200 µg PD-1 mAb in 300 µL PBS on days 6, 9, and 12 post-tumor implantation. After 15 days of this administration, the mice were sacrificed, and the solid tumors were excised and weighed.

### Antibiotic cocktail

The mice were treated with antibiotics 2 weeks before tumor implantation and continued until the end of the experiment. A mixture of vancomycin (500 mg/L), ampicillin (1 g/L), neomycin (1 g/L), and metronidazole (1 g/L) were added in sterile drinking water. Solutions were changed three times a week, and care was taken to avoid light. Antibiotic activity was analyzed by cultivating the fecal pellets of the mice resuspended in normal saline on LB solid culture plates for 24 h at 37 °C with 5% CO_2_ for aerobic conditions.

### Western blotting

The isolated tumor tissues were homogenized after adding the appropriate amount of RIPA Lysis Buffer and PMSF, and then centrifuged (12,000 × *g*, 4 °C) for 10 min to obtain the supernatant. Equal amounts of lysate proteins (20 μg) were electrophoretically separated by sodium dodecyl sulfate-polyacrylamide gel electrophoresis and transferred to polyvinylidene fluoride membranes. Membranes were blocked in Tris-buffered saline with Tween 20 (TBST) supplemented with 5% bovine serum albumin (BSA) at room temperature for 2 h and incubated at 4 °C overnight with primary antibodies. They were then subjected to HRP-conjugated secondary antibodies at room temperature for 1.5 h and proteins were detected using enhanced chemiluminescence system (Pierce, Rockford, IL, USA). Intensities in the resulting bands were quantified by ImageJ software.

### TUNEL assay

The isolated tumor tissues were fixed with 4% paraformaldehyde for at least 24 h, embedded in paraffin and sectioned. Then, paraffin sections were subjected to deparaffinization, rehydration, and permeabilization with proteinase K for 30 min. After being washed three times with PBS, the tumor tissue sections were treated with TUNEL Assay kit following the manufacturer’ s instruction. Samples were counterstained with hematoxylin after DAB coloration, dehydrated with xylene and then mounted. The apoptosis of tumor cells was visualized by fluorescence microscopy (Nikon, Japan).

### Histology and immunohistochemistry

Histological observation of tumor tissues was applied to examine the tumor necrosis. Paraffin sections of tumor tissues were deparaffinized and rehydrated, and then stained routinely with Harris hematoxylin and eosin.

For immunohistochemistry, the antigen of tumor paraffin sections was retrieved in EDTA (PH = 9) Antigen Repair Buffer. To quench the endogenous peroxidase activity, the samples were incubated with 3% H_2_O_2_ for 30 min and followed by blocking in 3% BSA in PBS. The slides were covered with correlative primary antibodies and incubated at 4 °C overnight, followed by incubation with secondary antibody at room temperature for 50 min. Then, the tissue sections were counterstained with hematoxylin after DAB coloration and mounted. All the samples were detected by an inverted microscope (Nikon, Japan).

### Flow cytometry

After the planned treatment, the isolated tumor tissues were filtered through 70 μm cell strainer (BD Falcon, USA) to obtain the single cells. The harvested cells were washed twice with PBS, and then stained in the dark with anti-mouse antibodies for CD3e, CD4, and CD8a at 4 °C for 30 min. Analysis was performed immediately by using an FACS Calibur flow cytometer (Becton-Dickinson, Fullerton, CA, USA).

### Bacterial identification in fecal samples

The C57BL/6 mice, randomized into two groups, were treated with diosgenin (20 mg/kg) and normal saline (control) by gavage everyday respectively. After 2 weeks of this administration, the feces of mice were collected and frozen at −80 °C.

Bacterial DNA from the feces of mice was extracted using MoBio PowerSoil® DNA Isolation Kit and subjected to PCR amplification. The highly variable V4 region of the bacterial 16S rRNA (about 280 bp) was used for sequencing to analyze microbial diversity. The PCR products were quantified on Microplate reader (BioTek, FLx800) by using Quant-iT PicoGreen dsDNA Assay Kit, and then mixed according to the amount of data required for each sample. Thereafter, the DNA library was constructed by using Illumina’s TruSeq Nano DNA LT Library Prep Kit. After quality control, sequencing, and quantitation of the library, MiSeq Reagent Kit V3 (600 cycles) was used for double-end sequencing of 2×300 bp on the MiSeq machine.

According to the quality of the sequences, the preliminary screening was conducted on the original sequencing data. The high-quality sequences were clustered into OTUs at 97% of identity by using QIIME software. Representative sequences of each OTU were used for taxonomic identification and phylogenetic analysis. Based on the abundance distribution of OTUs in different samples, the diversity of each sample was assessed. Subsequently, the specific composition of each sample at different classification levels was analyzed.

### Statistical analysis

The statistical analysis was performed by GraphPad Prism 5 with one-way ANOVA or two tailed Student’ s *t* test. All data were presented as mean ± SD and values of *P* *<* 0.05 were considered to be statistically remarkable.
